# Supplementary figures and images for: Anorectal Malformations (ARM) and associated maternal factors among children at Tikur Anbessa Specialized Hospital and St. Paul’s Hospital Millennium Medical College, Addis Ababa, Ethiopia: An unmatched case-control study
Source: PLoS One. 2024 Sep 20;19(9):e0309298. doi: 10.1371/journal.pone.0309298 (PMC11414888; doi:10.1371/journal.pone.0309298)

**S1 File. Sampling procedure.** Schematic representation of sampling procedure.

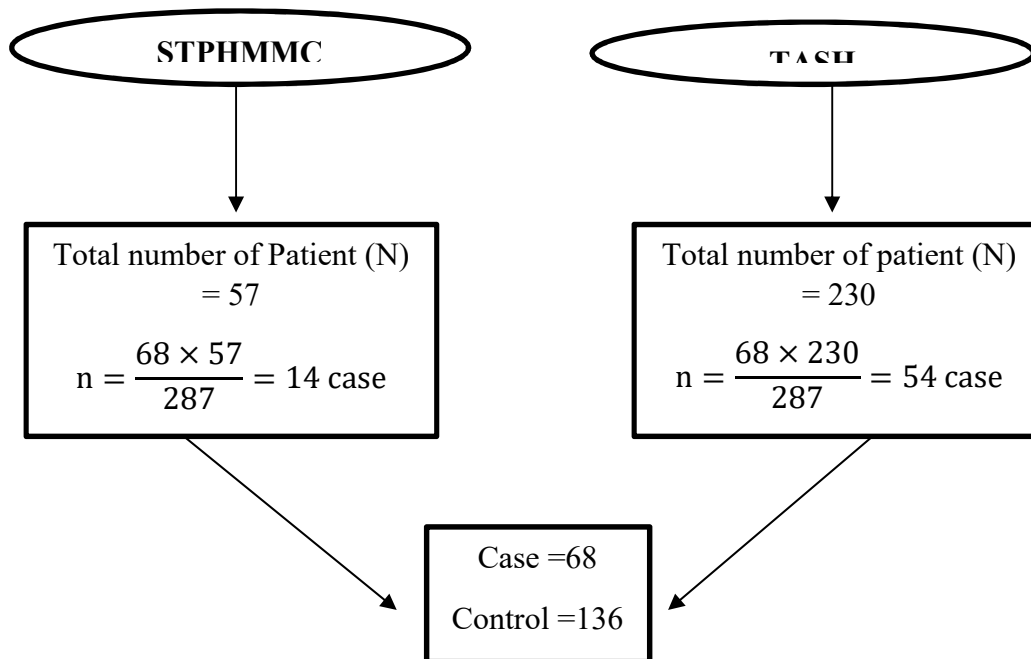

Supplement: S1 File — (PDF) [file pone.0309298.s001.pdf]
