# Supplementary material for: Anorectal Malformations (ARM) and associated maternal factors among children at Tikur Anbessa Specialized Hospital and St. Paul’s Hospital Millennium Medical College, Addis Ababa, Ethiopia: An unmatched case-control study
Source: PLoS One. 2024 Sep 20;19(9):e0309298. doi: 10.1371/journal.pone.0309298 (PMC11414888; doi:10.1371/journal.pone.0309298)
Supplement: S2 File — (DOCX) [file pone.0309298.s002.docx]

**Annex I: Consent form and questionnaire (English and Amharic Version)**

**ADDIS ABABA UNIVERSITY**

**COLLEGE OF HEALTH SCIENCES**

**SHOOL OF MEDICINE**

### **Mother Consent Form**

The study focuses to assess, risk factors associated with anorectal malformation, in selected governmental hospitals in Addis Ababa, Ethiopia.

Good morning /Afternoon/ my name is ‑‑‑‑‑‑‑‑‑‑‑‑‑‑‑. I came from Addis Ababa University, collage of health science, school of medicine, department of Medical Anatomy. The principal investigator doing this thesis is for the partial fulfillment of masters’ degree of Medical Anatomy. The name of the principal investigator of this study is Ms Samrawit Solomon. Now we would like to ask you few questions.

You are selected and included in the study as part of the sample population to complete the questionnaire designed by the researcher because you fulfill requirement for sampling. Thus this interview is prepared for this purpose to get appropriate data on the study I am conducting. The data that I will obtain using this interview will be used only for research purpose and your response will be kept confidential. For this purpose, your name will not be written here and there will be no way of linking your individual responses to the result of the study findings.

The study has no risk to you and your family except taking a maximum of 30 minutes of your time and if you face any problem in relation to the research, you can contact responsible person based on the address below. You have the right not to respond at all or to withdraw in the meantime, but your participation is highly valuable for the success of my research objectives. Therefore, I kindly request your cooperation to participate in this interview with a great respect.

Do you agree to participate in this study?

Yes, (Continue) ------ No, (stop) -------.

Thanks a lot!

አዲስ አበባ ዩኒቨርሲቲ

የጤናሳይንስ ኮሌጅ

የስምምነት ፎርም

የአብሮ ወለድ ጉድለቶችና ተያያዥየ ሆኑ አጋላጭ ምክንያቶች ጥናት በአዲስ አበባ ከተማ ውስጥ በሚገኙ መንግሥታዊ ሆስፒታሎች

ጥናቱ የሚያተኩረው በአዲስ አበባ፣ ኢትዮጵያ በተመረጡ የመንግስት ሆስፒታሎች ውስጥ፣ ከአኖሬክታል መዛባት ጋር ተያይዘው የሚመጡ አደጋዎችን ለመገምገም ነው።

እንደምን አደሩ ስሜ __________________ እባላለሁ:: የመጣሁት ከአዲስ አበባ ዩኒቨርሲቲ፣ ከጤና ሳይንስ ኮሌጅ፣ ከሕክምና ትምህርት ቤት፣ ነው። ይህንን ጥናት የሚያካሂደው ዋናው መርማሪ የሕክምና አናቶሚ የማስተርስ ዲግሪ በከፊል ለማሟላት ነው። የዚህ ጥናት ዋና መርማሪ ስም ወ/ሮ ሳምራዊት ሰለሞን ይባላሉ።

በተመራማሪው የተነደፈውን መጠይቅ ለመሙላት የናሙና ህዝብ አካል በመሆን ተመርጠው በጥናቱ ውስጥ ተካተዋል ምክንያቱም የናሙና መስፈርቶችን ስላሟሉ ነው። ስለዚህ ይህ ቃለ መጠይቅ እኔ በምመራው ጥናት ላይ ተገቢውን መረጃ ለማግኘት ለዚህ ዓላማ ተዘጋጅቷል። ይህንን ቃለ መጠይቅ ተጠቅሜ የማገኘው መረጃ ለምርምር ዓላማ ብቻ የሚውል ሲሆን ምላሽህ በሚስጥር ይጠበቃል። ለዚሁ ዓላማ፣ ስምዎ እዚህ አይጻፍም እና የግል ምላሾችዎን ከጥናቱ ግኝቶች ጋር የሚያገናኙበት መንገድ አይኖርም።

ጥናቱ ቢበዛ 30 ደቂቃ ከመውሰድ በቀር ለእርስዎና ለቤተሰብዎ የሚያስከትለው ምንም አይነት አደጋ የለውም እና ከጥናቱ ጋር በተያያዘ ምንም አይነት ችግር ካጋጠመዎ ከዚህ በታች ባለው አድራሻ ተጠያቂውን ሰው ማነጋገር ትችላለ። እስከዚያው ድረስ ምንም አይነት ምላሽ የመስጠት ወይም የመውጣት መብት አልዎት፣ ነገር ግን የእርስዎ ተሳትፎ ለምርምር አላማዬ ስኬት ከፍተኛ ዋጋ ያለው ነው። ስለሆነም በዚህ ቃለ ምልልስ ላይ እንድትሳተፉ ከታላቅ አክብሮት ጋር እንድትተባበሩ በትህትና እጠይቃለሁ።

በዚህ ጥናት ለመሳተፍ ተስማምተሃል?

1. አዎ፣ (ቀጥል) ------
2. አይ፣ (አቁም) -------.

እናመሰግናለን!
