# Supplementary material for: Anorectal Malformations (ARM) and associated maternal factors among children at Tikur Anbessa Specialized Hospital and St. Paul’s Hospital Millennium Medical College, Addis Ababa, Ethiopia: An unmatched case-control study
Source: PLoS One. 2024 Sep 20;19(9):e0309298. doi: 10.1371/journal.pone.0309298 (PMC11414888; doi:10.1371/journal.pone.0309298)
Supplement: S3 File — (PDF) [file pone.0309298.s003.pdf]

Addis Ababa University

College of health science, school of medicine

Department of human anatomy

**Data Collecting Format for the assessment of risk factors associated with ARM of Anorectal malformation at selected governmental hospitals in Addis Ababa, Ethiopia, 2023**

**Code ----------------------**

**MRN-----------------------**

**Case/Control--------------**

| **Socio-demographic characteristics of the mother** | | |
|---|---|---|
| **1** | Age of a mother at pregnancy | ______________________________ |
| **2** | Residency | 1.urban<br>2.rural |
| **3** | Region | 1.Addis Ababa<br>2.Oromia<br>3.Amhara<br>4.Somalia<br>5.Benshangul Gumuz<br>6.Dire Dewa<br>7.Harari<br>8.Tigray<br>9.Other (specify)--------------- |
| **4** | Religion | 1.Orthodox<br>2. Muslim<br>3.prothestant<br>4. Catholic<br>5.Other |
| **5** | Educational status | 1.Ilitrate<br>2.Can read and write<br>3.Elementary school<br>4. High school graduate<br>5.Higher education |

| | | |
|---|---|---|
| **6** | Occupation | 1.government<br>2. student<br>3.farmer<br>4.private<br>5.other<br>6.Specify ---------------- |

## Maternal reproductive and obstetric history

| No | **Question** | **Coding category** | **Skip** |
|---|---|---|---|
| **1** | The index pregnancy was | 1. single tone<br>2. multiple pregnancy | |
| **2** | Gravidity<br>Parity | -------------------------<br>------------------------- | |
| **3** | Gestation age at delivery | 1.Term<br>2. Preterm | |
| **4** | Birth weight of child | -------------------------- | |
| **5** | Did you have any of this in your previous pregnancies? | 1.Abortion<br>2.Still birth<br>3.Early neonatal death<br>4. More than one specify | |
| **6** | Had ANC follow up? | 1 . yes ⇩ 2. No ⇨ | 8 |
| **7** | If yes how many follow-ups did you attend? | 1.1<br>2.2<br>3.3<br>4.4 | |
| **8** | Was the pregnancy planned | 1. yes 2. No | |

## Genetic Factor

| | | | |
|---|---|---|---|
| **1** | Previous history of congenital anomaly in the family? | 1.Yes ⇩ 2.No ⇨ | 4 |
| | How is that person related to this child? | 1.First degree relative<br>2.Second degree relative<br>3. Third degree relative | |
| **2** | Previous history of anorectal malformation in the family? | 1.Yes ⇩ 2.No ⇨ | 4 |
| **3** | How is that person related to this child? | 1.First degree relative<br>2.Second degree relative<br>3. Third degree relative | |
| **4** | Do you have genetically relationship with your husband? | 1 . yes 2. No | |

## Maternal Medical And Drug History

| | | | |
|---|---|---|---|
| **1** | Is there any history of maternal medical disease | 1. Yes 2. No ⇨ | 3 |
| **2** | What type of disease | 1.Diabetes mellitus<br>2.Anemia<br>3.Epilepsy<br>4.urinary tract infection<br>5. Other ------------------------ | |
| **3** | History of medication intake during the first trimester | 1. Yes 2. No ⇨ | Next part |
| **4** | What type of medication | --------------------------------- | |
| **6** | How long did she use it | ----------------------------------- | |

## Maternal nutritional and folic acid consumption

| | | | |
|---|---|---|---|
| **1** | History of folic acid intake during the pregnancy? | 1.Yes 2.. No ⇨ | 4 |
| **2** | If yes, when did she start using them? | 1. Before pregnancy (specify)……………………………… <br>2. After conceiving (specify gestation age)………………………… | |
| **3** | For how long did the mother use folic acid? | | |
| **4** | What was your weight and height at the beginning of pregnancy | weight (Kg)__________<br>height (meter)__________ | |

## Maternal Chemical Exposure and maternal lifestyle

| | | | |
|---|---|---|---|
| **1** | History of alcohol intake during the first trimester of pregnancy? | 1. Yes 2. No ⇨ | 4 |
| **2** | alcohol intake per day and how often | | |
| **3** | History of smoking cigarette during pregnancy? | 1. Yes 2. No ⇨ | 8 |
| **4** | the amount of cigarette per day | | |
| **5** | At which gestation age | | |
| **6** | Was the mother staying with the cigarette smoker during pregnancy | 1.Yes 2.No | |
| **7** | Was the mother exposed to theaputic/diagnostic radiation during pregnancy? | 1. Yes 2. No ⇨ | 11 |
| **8** | Type and gestation age of radiation exposures did she had? | ------------------------------ | |
| **9** | At what estimated gestation age | | |
| **10** | Have you used any pesticides (insecticides, herbicides or fungicides) at home /work place? | 1. Yes<br>2. No | |
| **11** | exposure to industrial cleaning agents and solvents | 1. Yes<br>2. No------------- | |
