# Supplementary material for: Anorectal Malformations (ARM) and associated maternal factors among children at Tikur Anbessa Specialized Hospital and St. Paul’s Hospital Millennium Medical College, Addis Ababa, Ethiopia: An unmatched case-control study
Source: PLoS One. 2024 Sep 20;19(9):e0309298. doi: 10.1371/journal.pone.0309298 (PMC11414888; doi:10.1371/journal.pone.0309298)
Supplement: Attachment — Submitted filename: Reviewers notes.docx [file pone.0309298.s004.docx]

**Reviewer’s Comment/ PLOS**

**General**: It is a relevant topic. It needs proper editing of language usage and for consistent use of terms, figures and flow of idea and avoid repetitive notes

1. **Abstract**

Settings: Not proper description of study area in methods section of the manuscript

ODK: It is not mentioned in the body (methods) of the manuscript

2. **Methods**
a. The methods section needs further reorganization

b. The methods section should clearly show the study area, period, settings, design, sampling technique (controls), variables of interest, data collection procedure, data quality assurance methods and analysis methods and only information included in this section can be reported in the abstract section.

c. Why were other congenital anomalies other than ARM were also excluded? (exclusion criteria should be described well)

d. What does non-ARM related disease mean?

e. Hospital based or population based control selected?

f. Inclusion of children: Is this reflected in the analysis?

g. What is the target population of study? Children with ARM or Mothers?

h. Mention as oral consent in other part of the study?

i. Why was 5% used as non-response mark than the usual 10%?

j. How was data quality control assured?

k. How was variable selection made for inclusion (p-values) in the multivariable analysis

l.  How was multicollinearity checked?

m. How was the final model of multivariable regression checked?

3. **Results**

a. How was the overall response rate?

b. Illness: Did you check separately or in groups for each disease entity for significance? (statistical significance might have been different)

c. What does unspecified medications mean?

d. How was it different with different groups of illness analyzed above? (statistical significance might have been different)

e. Which trimester of pregnancy was of interest in data collection? Was the pre-pregnancy period considered? (mainly for medication, alcohol and smoking)

f. Was maternal smoking status assessed other than passive smoking?

g. Redundant paragraph information

h. Check again, some of the %s do not add up to 100% on tables

i. Check again for chi-square assumption in each cell; some of the cells need regrouping for analysis

4. **Discussion**
a. Only results addressed in the results section and pertinent factors should be included in the discussion
b. General terms should be avoided like, sampling technique, design etc in the discussion section on result alignment or disparity
c. **Conclusion**

Re-write focused to the study findings; pertinent to the study results, future suggestions, limitations of the study should be raised here
