# Supplementary material for: Anorectal Malformations (ARM) and associated maternal factors among children at Tikur Anbessa Specialized Hospital and St. Paul’s Hospital Millennium Medical College, Addis Ababa, Ethiopia: An unmatched case-control study
Source: PLoS One. 2024 Sep 20;19(9):e0309298. doi: 10.1371/journal.pone.0309298 (PMC11414888; doi:10.1371/journal.pone.0309298)
Supplement: Attachment — Submitted filename: Reviewers notes on document (1).docx [file pone.0309298.s005.docx]

# PLOS ONE

**Maternal risk factors associated with Anorectal malformation at selected governmental**

**hospitals in Addis Ababa, Ethiopia, 2023: A case–control study**

--Manuscript Draft--

| **Manuscript Number:** | PONE-D-24-13124 |
| --- | --- |
| **Article Type:** | Research Article |
| **Full Title:** | Maternal risk factors associated with Anorectal malformation at selected governmental hospitals in Addis Ababa, Ethiopia, 2023: A case–control study |
| **Short Title:** | Risk factors associated with Anorectal malformation |
| **Corresponding Author:** | Solomon Tibebu, BSc., MD, MSc  Addis Ababa University Faculty of Medicine: Addis Ababa University School of Medicine  Addis Ababa, ETHIOPIA |
| **Keywords:** | Anorectal malformation; ARM; Risk factors; Tikur Anbessa Specialized Hospital; Saint Pauli's Hospital Millennium Medical College. |
| **Abstract:** | Introduction: Anorectal malformations are a wide spectrum of congenital defects involving the lowest portion of the intestinal and urogenital tracts. It occurs between six weeks and tenth week of the embryonic development. Although the exact cause of anorectal malformation are still mostly unclear, a complex etiology involving interaction between genetics and environment is thought to be responsible.  Objective: The purpose of this study is to assess maternal risk factor associated with the occurrence of anorectal malformation at selected governmental hospitals in Addis Ababa, Ethiopia 2023.  Methods: An unmatched case-control study was conducted from August 2022 to January 2023in Addis Ababa Ethiopia involving ARM children and their mothers as cases and children without ARM and their mothers as controls who were admitted to Tikur Anbessa Specialized Hospital and St Paul's Hospital Millennium Medical College. The sample size was determined by Epi Info 7 statistical software packages.  The data was collected by structured questionnaire using ODK and then exported into SPSS version 26 for management and analysis.  Both descriptive and analytical statistics was used. Bivariate and multivariate binary logistic regressions analysis was carried out to determine the association of independent variables with Anorectal malformation. In a multivariable model, an adjusted odds ratio (AOR) with a 95% confidence interval was computed and the statistical significance was declared at a p-value < 0.05.  Result: The present study showed the presence of a statistically significant association between advanced maternal age (age > 35 years) and birth of newborns with ARM. According to the study, mothers with advanced age were 4.2 times more likely to give birth to newborns with ARM than younger maternal age (AOR = 4.222; 95% CI: 1.214- 14.689).  Mothers with first and second generation family history of birth defect were six times more likely to have a child with Anorectal malformation compared to their counterparts (AOR = 6.15; 95% CI: 1.237, 30.582).  In addition, the likelihood of having newborns with Anorectal malformation was five times higher for women who had a history of alcohol consumption and taking unspecified medication during the first trimester of the pregnancy compared to their counterparts (AOR = 4.707; 95% CI: 1.705-12.97) and (AOR = 4.857; 95% CI: 1.288-  18.318) respectively.  Furthermore, low birth weight and unplanned pregnancy were also significantly associated with the occurrence of Anorectal malformation (AOR = 4.318; 95% CI: 1.199, 15.551) and 3.701; 95% CI: 1.551-8.828 respectively).  Conclusion: In conclusion, advanced maternal age, unspecified medication intake in the first trimester of pregnancy, low birth weight, and alcohol consumption, family history of congenital malformations and unplanned pregnancy appeared to increase the risk of Anorectal malformation. |
| **Order of Authors:** | Samrawit Solomon, BSc; MSC |
|  | Fisseha Temesgen, MD, Subspeciality in pediatric Surgery |

|  | Solomon Tibebu, BSc., MD, MSc |
| --- | --- |
|  | Hana Abebe, MD, Subspeciality in Pediatric Surgery |
|  | Girma Seyoum, Ph.D |

### Maternal risk factors associated with anorectal malformation at selected governmental Hospitals in Addis Ababa, Ethiopia, 2023: A case–control study

Abstract

### Introduction

Anorectal malformations are a wide spectrum of congenital defects involving the lowest portion of the intestinal and urogenital tracts. It occurs between six weeks and tenth week of the embryonic development. Although the exact cause of anorectal malformation are still mostly unclear, a complex etiology involving interaction between genetics and environment is thought to be responsible.

### Objective

The purpose of this study is to assess maternal risk factor associated with the occurrence of anorectal malformation at selected governmental hospitals in Addis Ababa, Ethiopia 2023.

### Methods

An unmatched case-control study was conducted from August 2022 to January 2023in Addis Ababa Ethiopia involving ARM children and their mothers as cases and children without ARM and their mothers as controls who were admitted to Tikur Anbessa Specialized Hospital and St Paul's Hospital Millennium Medical College.

The sample size was determined by Epi Info 7 statistical software packages.

The data was collected by structured questionnaire using ODK and then exported into SPSS version 26 for management and analysis.

Both descriptive and analytical statistics was used. Bivariate and multivariate binary logistic regressions analysis was carried out to determine the association of independent variables with Anorectal malformation. In a multivariable model, an adjusted odds ratio (AOR) with a 95% confidence interval was computed and the statistical significance was declared at a p-value < 0.05.

### Result

The present study showed the presence of a statistically significant association between advanced maternal age (age > 35 years) and birth of newborns with ARM. According to the study, mothers with advanced age were 4.2 times more likely to give birth to newborns with ARM than younger maternal age (AOR = 4.222; 95% CI: 1.214-14.689).

Mothers with first and second generation family history of birth defect were six times more likely to have a child with Anorectal malformation compared to their counterparts (AOR = 6.15; 95% CI: 1.237, 30.582).

In addition, the likelihood of having newborns with Anorectal malformation was five times higher for women who had a history of alcohol consumption and taking unspecified medication during the first trimester of the pregnancy compared to their counterparts (AOR = 4.707; 95% CI: 1.705-12.97) and (AOR = 4.857; 95% CI: 1.288-18.318) respectively.

Furthermore, low birth weight and unplanned pregnancy were also significantly associated with the occurrence of Anorectal malformation (AOR = 4.318; 95% CI: 1.199, 15.551) and 3.701;

95% CI: 1.551-8.828 respectively).

### Conclusion

In conclusion, advanced maternal age, unspecified medication intake in the first trimester of pregnancy, low birth weight, and alcohol consumption, family history of congenital malformations and unplanned pregnancy appeared to increase the risk of Anorectal malformation.

Keywords: Anorectal malformation; ARM; Risk factors; Tikur Anbessa Specialized Hospital; Saint Pauli’s Hospital Millennium Medical College.

# Introduction

Anorectal malformation (ARM) is a spectrum of congenital anomaly involving the anorectum and variable portions of the urogenital tract. It occurs as a result of an abnormal development of the terminal portion of the genitourinary tract occurring during the early intrauterine life, between 6 weeks and tenth week of fetal development (1,2). Depending on the level of disturbance, they carry a spectrum of malformation ranging from minor skin level defects such as rectoperineal or anoperineal fistulas to severe and complex forms such as persistent cloaca (3, 26).

The birth prevalence of ARM ranges from 1 in 1500 to 1 in 5000 live births worldwide with geographical and yearly/seasonal variations (4). Many literatures in Africa identify anorectal malformation as the commonest cause of neonatal gastrointestinal obstruction and also the leading congenital pediatric surgery cases in Africa (1). In Ethiopia 56 % of early neonatal intestinal obstruction is caused by ARM (5). The etiology of ARM is largely unknown. In rare syndromic types such as Townes–Brocks syndrome, Down syndrome and Currarino syndrome, the cause of ARM can be explained by genetic factors mainly chromosomal abnormalities. However, the majority of ARM cases are non-syndromic and their cause is assumed to be multifactorial due to interaction between genetics and the environment (7). Environmental risk factors like maternal smoking, maternal obesity, maternal diabetes, multiple pregnancy, previous miscarriage, maternal chronic disease were described as risk factors for ARM according to different literatures (8, 9).

In this study, we investigated the associated risk factors of ARM among patients who presented to selected governmental hospitals, Addis Ababa, Ethiopia, as there are no literatures regarding the associated risk factors of ARM in the population of Ethiopia.

## METHODS AND materials

This study was conducted at Tikur Anbessa Specialized Hospital (TASH) and St Paul's Hospital Millennium Medical College (SPHMMC), a governmental Territory hospitals located in Addis Ababa Ethiopia from August 2022-January 2023 G.C.

The study used case control study design. Cases were consecutive mothers of all infants with ARM and controls were mothers of infants without any congenital anomaly and came for treatment of other non-ARM related diseases during the study period (August 2022-January 2023 G.C). All children diagnosed with ARM and who meet the inclusion criteria were included in the study.

*Operational definitions cases and controls should be part of the operational definition* Anorectal malformations were defined as the absence of anus or abnormal anus with or without a fistula and they were classified into different subgroups according to Krickenebeck criteria^25^

Data were obtained from hospital medical records and questionnaire surveys that were completed after participant gave their written concent to participate in the study.

Sample size was calculated using Epi Info version, Stat Calculator considering 95% confidence level (CI), 80% power, control to case ratio of 2:1 and Family history of ARM from similar study which gives large sample size compared to other exposure leads to estimate case exposure status 7.2% and control exposure status 0.2 %.

By considering 5% non-responding rate, the calculated sample was 68 cases and 136 controls and a total of 204 sample size (9).

The data was entered and analyzed using Statistical Package Social Sciences (SPSS) version 26 software.

Both descriptive and analytical statistics was used. Bivariate and multivariate binary logistic regressions analysis was carried out to determine the association of independent variables with ARM. In a multivariable model, an adjusted odds ratio (AOR) with a 95% confidence interval was computed and the statistical significance was established at p-value </= 0.05.

## RESULTS

### Socio-demographic characteristics

A total of 204 (68 cases and 136 controls) mothers were identified and interviewed at the pediatrics inpatient and outpatient units. About 87.5% of mothers of controls and more than half of mothers of cases (76.4%) were in the age group of 20–34 years. The majority of women (54.4% of cases and 57.3% of controls) were orthodox in religion followed by Muslim (33.8% of cases and 30.9%).

Regarding educational status, of the mothers 35.3% of cases and 39.7% of controls were high school graduates. Concerning the residency of the mothers 67.6% of cases and 94.8% of controls were urban dwellers where 38.2% of the case and 60.3% controls were from Addis Ababa. Majority of the mothers were housewives (69.8% of cases and 57.3% of controls) (Table 1).

### Reproductive, obstetric and other risk factors of ARM

In our study, the majority of the women (79.4% of cases and 61% of controls) were multiparous,

35.3. About one percent of (1%) of the controls and 29.4% of cases gave birth for the second time.

The present study showed the presence of a statistically significant association between advanced maternal age (age > 35 years) and birth of newborns with ARM. According to the study, mothers with advanced age were 4.2 times more likely to give birth to newborns with ARM than younger maternal age (table 2).

Nearly half of pregnancy (44.1%) in the ARM groups and 13.2% of pregnancies in control group were not planned. This finding indicates that there was highly statistically significant difference between the two groups (P= 0.003).

Eighty-nine percent of mothers in both cases and controls gave birth at 37 weeks and above. Out of the total number mothers in ARM group and control groups only 4.4 % and 7.3% gave birth prior to 37 weeks of gestation respectively. About 12% of mothers in ARM group and 5.9% of mothers in the control group had abortion history. However, the difference was not statistically significant.

Regarding family history, out of the total 68 ARM groups, 9(12.2%) and 136 control groups 4 (2.9%) had a family history congenital anomalies which was found to be a statistically significant difference (p=0.026). In addition, there was no single newborn in the control group with family history of ARM while there were two newborns (2.9%) in the ARM group with family history of ARM.

Although not statistically significant, the present study revealed that maternal medical illnesses such as Urinary tract infection, anemia and hypertension, Diabetes mellitus and Asthma occurred more often in ARM groups than control groups which was 44% and 16% respectively.

In terms of unspecified medication use, there was statistically significant difference (p<0.05) between cases or ARM groups 22.1% (15/68) and controls groups 5.9% (8/136).

Out of the total controls, 93(68.4%) and total cases, 40(59%) had normal body mass index in the early pregnancy. Twenty-eight percent of the cases and 24% of the controls were overweight.

There was statistically significant association between Alcohol consumption during pregnancy and birth of newborns with ARM. Mothers who drink alcohol had 4.7 times higher risk having newborns with ARM than mother who had no history of alcohol consumption (p=0.003)(Table 2).

Passive smokers were observed only in 4 (5.9%) of cases and 1(0.7%) of controls.

About 12(17.6%) of case mothers and 4(2.9) % of control mothers were exposed to chemicals like pesticides at the time of pregnancy. Exposure to industrial cleaning agents was seen in 7(10.3%) of case mothers and 9(6.6%) of control mothers. Likewise, the likelihood of having newborns with ARM was five times higher for women who had a history of taking unspecified medication during the first trimester of the pregnancy (AOR = 4.857; 95% CI: 1.288, 18.318 ).

Table 1: Socio-demographic characteristics of cases and controls treated at selected Governmental Hospitals Addis Ababa, Ethiopia 2023.

| **Characteristics** | | **Case** | | **Controls** | |
| --- | --- | --- | --- | --- | --- |
| Mother Age | category | Frequency | % | Frequency | % |
|  | <20 years | 0 | 0 | 10 | 7.3 |
|  | 20 – 34 years | 52 | 76.5 | 119 | 87.5 |
|  | >34 years (advanced  maternal age) | 16 | 23.5 | 7 | 5.1 |
| Child Mother Education | Illiterate | 22 | 32.4 | 3 | 2.2 |
|  | Can read and write | 3 | 4.4 | 14 | 10.3 |
|  | Elementary education | 10 | 14.7 | 36 | 26.5 |
|  | High school education | 24 | 35.3 | 54 | 39.7 |
|  | Higher education | 9 | 13.2 | 29 | 21.3 |
| Child Mother Occupation | Housewife | 39 | 57.3 | 95 | 69.8 |
|  | Student | 1 | 1.5 | 2 | 1.5 |
|  | Government employee | 13 | 19.1 | 15 | 11 |
|  | Private | 10 | 14.7 | 22 | 16 |
|  | Farmer | 5 | 7.4 | 2 | 1.5 |
| Region of the mother | Addis Ababa | 26 | 38.2 | 82 | 60.3 |
|  | Oromia | 30 | 44.1 | 33 | 24.3 |
|  | Amhara | 8 | 11.8 | 11 | 8.1 |
|  | SNNP | 3 | 4.4 | 7 | 5.1 |
|  | Afar | 1 | 1.5 | 0 | 0 |
|  | Somalia | 0 | 0 | 3 | 2.2 |
| Parity | Multi | 54 | 79.4 | 83 | 61 |
|  | One | 14 | 20.6 | 53 | 39 |
| Gestational age at delivery | Pre-term | 3 | 4.4 | 10 | 7.3 |
|  | Term | 61 | 89.7 | 122 | 89.7 |
|  | Post term | 4 | 5.9 | 4 | 3 |
| Birth weight of a child | Very low birth weight | 0 | 0 | 0 | 0 |
|  | Low birth weight | 11 | 16.3 | 8 | 5.9 |
|  | Normal birth weight | 54 | 79.4 | 128 | 94.1 |
|  | Macrosomic | 3 | 4.4 | 14 | 10.3 |
| BMI | Under weight | 5 | 7.3 | 7 | 5.1 |
|  | Normal | 40 | 59 | 93 | 68.4 |
|  | Over weight | 19 | 28 | 33 | 24.3 |
|  | Obese | 4 | 5.8 | 3 | 2.2 |

Table 2: Binary and multinomial logistic regression of risk factors for ARM at selected governmental hospitals Addis Ababa, Ethiopia 2023.

| Characteristic  S |  | Case (N,  %) | Control (N,  %) | COR (95% CI) | AOR (95% CI) | P-value |
| --- | --- | --- | --- | --- | --- | --- |
| ANC follow up | Yes | 60(88.2) | 132 (97.1) | 0.0.227(0.066,0.  784) | 0.742(0.120,  4.593) | 0.744 |
|  | No | 8(11.8) | 4 (2.9) | 1 | 1 |  |
| Advanced maternal age | Yes | 16(23.5) | 7 (5.1) | 5.670(5.670,14.5  85) | 4.222(1.214,  14.689) | 0.024* |
|  | No | 52(76.5) | 129 (94.9) | 1 | 1 |  |
| Low  Birth weight | Yes | 11(16.2) | 8 (5.9) | 3.088(1.179,  8.086) | 4.318(1.199,  15.551) | 0.025* |
|  | No | 57  (83.8) | 128 (94.1) | 1 | 1 |  |
| History of Maternal medical  Illness | Yes | 30(44.1) | 22(16.2) | 4.091(2.111,  7.926) | 2.265(.894, 5.718) | 0.085 |
|  | No | 38(55.9) | 114(83.8) | 1 | 1 |  |
| Unspecified medication  Use | Yes | 15(22) | 8(5.9) | 4.528(1.812,  11.361) | 4.857(1.288,  18.318) | 0.02* |
|  | No | 53(78) | 128(94.1) | 1 | 1 |  |
| Passive smoking | Yes | 9(12.2) | 4(2.9) | 5.034(1.490,  17.002) | 6.150(1.237,  30.582) | 0.026* |
|  | No | 59(86.8) | 132(97.1) | 1 | 1 |  |
| Exposure to Pesticide | Yes | 12(17.6) | 4(2.9) | 9.500(2.581,  34.967) | 2.526(0.495,  12.896) | 0.265 |
|  | No | 56(82.4) | 132(97.1) | 1 | 1 |  |
| Folic acid intake in the  First trimester | Yes | 53(77.9) | 72(52.9) | 3.141(1.616,  6.106) | 2.309(0.983,  5.420) | 0.055 |
|  | No | 15(22.1) | 64(47.1) | 1 | 1 |  |
| Unplanned pregnancy | Yes | 30(44.1) | 18(13.2) | 5.175(2.598,  10.311) | 3.701(1.551,  8.828) | 0.003* |
|  | No | 38(55.9) | 118(86.7) | 1 | 1 |  |
| History of abortion | Yes | 12(17.6) | 8(5.9) | 3.429(1.328,  8.086) | 1.442(.441, 4.722) | 0.545 |
|  | No | 54(79.4) | 128(94.1) | 1 | 1 |  |
| Alcohol consumption | Yes | 21(30.9) | 13(9.6) | 4.617(2.107,10.1  19) | 4.707(1.705,12.99  7) | 0.003* |
|  | No | 47(69.1) | 123(90.4) | 1 | 1 |  |

Note: COR: Crude odds ratio, AOR: Adjusted odds ratio, CI: Confidence interval, * indicates statistical

## DISCUSSION

This study was conducted to determine the maternal factors associated with ARM in Addis Ababa, Ethiopia. The cause of ARM is not fully understood. However, existing literature suggests that it is multifactorial condition, involving both genetic and environmental factors play a role in its pathogenesis (7).

Genetic factors have been highlighted in many studies on ARM, including a study conducted in the Netherlands, Germany which found a higher risk of ARM in individuals with a family history of birth defects, including ARM, in their first and second-degree relatives (9-11). The present study is in agreement with these findings. We also found that individuals with a family history of birth defects, including ARM in their first and second-degree relatives, were six times more likely to develop ARM than those without such a family history.

Several environmental factors have been investigated in studies on the etiology and risk factors of ARM, but the results have been inconsistent and heterogeneous. Our research suggests that maternal alcohol consumption during early pregnancy may increase the risk of ARM, which is consistent with studies conducted in Japan on patients with ARM and Ethiopia on congenital anomalies (14, 15). This may be due to the fact that alcohol exposure can disrupt normal embryonic development processes such as cell proliferation, migration, and differentiation by crossing the placental membrane (ref).

However, studies conducted in Germany, the Netherlands, and Sweden has not found a significant association between alcohol consumption and the occurrence of ARM (10, 11, 16, 17). One possible explanation for these conflicting results is that the effects of alcohol on embryonic development are dependent on the dose, timing, duration, and pattern of exposure. Additionally, cultural differences in alcohol consumption patterns in different communities may also play a role. For example, in Ethiopia, locally made alcoholic beverages such as "areki" and "tella" are consumed on daily base by both men and women, particularly in rural communities (14).

This study identified medication use during the first trimester of pregnancy as another potential risk factor for ARM. This finding is consistent with other studies, including a meta-analysis on maternal drug use and ARM occurrence, which found associations with various drug groups such as anti-asthma, anti-pain, and anti-depressant medications, as well as thyroid medication and other drug groups (18). A research study conducted in Ethiopia on congenital anomalies also reported this association (14). However, some reports, such as those by Melanie Kapapaet al and Iris A. L. M. van Rooij, did not find a significant association between medication use during pregnancy and ARM (10, 12). The conflicting results in the literature may be due to various factors, such as differences in study design, sample size, and the specific medications investigated. (9).

The study found that patients with ARM had significantly lower birth weights compared to the control group. These results align with a previous study by Anna Svenningsson et al , which also found a significant association between low birth weight and the occurrence of ARM (17).

We found that both advanced maternal age was associated with a higher risk of developing ARM. Previous studies have also suggested that older mothers have a higher risk of genetic mutations and an increased incidence of aneuploidy, which could potentially contribute to the development of ARM. (21, 22).

The study revealed that unplanned pregnancy was also associated with high risk of developing ARM. Previous studies have shown that unplanned pregnancies are significantly associated with adverse birth outcomes but there was no clear evidence to suggest that unplanned pregnancy directly causes an increased risk of ARM. The possible explanation could be unplanned pregnancy can indirectly contribute to the risk of ARM by increasing the likelihood of certain risk factors that have been associated with the condition, such as low birth weight. Additionally, unplanned pregnancies may be associated with a lack of access to prenatal care, which can lead to undetected and untreated issues that could potentially contribute to the development of ARM or other birth defects (23).

The study found that several factors, including maternal BMI, maternal medical illness during pregnancy (such as diabetes, anemia, hypertension, and urinary tract infection), folic acid intake, maternal smoking, and passive smoking, did not have a significant association with the occurrence of anorectal malformations (ARM). This contrasts with previous studies conducted in Germany, the Netherlands, Sweden, China, and Japan, where different variables were found to be associated with ARM in different areas, resulting in heterogeneous results (10, 11, 15, 17, and 24).

The variation in results across different studies may be due to differences in study design, such as variations in the definition and classification of ARM, sample size, recruitment strategies, and methods used to collect and analyze data. In addition, differences in healthcare practices and access to prenatal care may also contribute to heterogeneity in the results. For example, limited access to prenatal screening and diagnostic tests in some areas of the world could affect the detection and reporting of ARM cases. Furthermore, differences in cultural and socioeconomic factors may also play a role in the heterogeneity of results. For instance, cultural practices or beliefs may influence the reporting or diagnosis of ARM cases, and socioeconomic factors like poverty or lack of access to healthcare may increase the risk of ARM (9).

CONCLUSION

Several studies were conducted on the risk factors of ARM. However, these studies are inconsistent and heterogeneous. In this study it was found that advanced maternal age, unspecified medication intake in the first trimester of pregnancy, low birth weight, alcohol consumption during the first trimester of pregnancy, a family history of congenital malformations and unplanned pregnancy appeared to increase the risk of ARM. Some of the expected risk factors like maternal illness including DM and maternal smoking were not found to be risk factors for ARM in our study, whereas unintended pregnancy was found to be a risk factor in our study.

Abbreviations

AAU: Addis Ababa University, ARM: Anorectal Malformation, TASH: Tikur Anbessa Specialized Hospital, STPHMMC: Saint Pauli’s Hospital Millennium Medical College

Acknowledgements

We would like to express our deepest gratitude to all the staffs of department of pediatrics surgery, Addis Ababa University and STPHMMC for their support.

Funding

This research received no specific grant from any funding agency

DECLARATION

ETHICAL APPROVAL AND CONCENT TO PARTICIPATE

This study was carried out after Ethical approval letter was obtained from Department of Anatomy Research Ethics Review Committee (DRERC), Addis Ababa Public Health Research and Emergency Management Directorate and Institute of Review Board (IRB) of Saint Pauli’s Hospital Millennium Medical College.

Consent for publication Not applicable

Competing interests: The authors declare that they have no conflicts of interest.

Corresponding author: Solomon Tibebu, Department of Anatomy, School of Medicine, college of health sciences, Addis Ababa University, Ethiopia. E-mail: [solomonti04@yahoo.com](mailto:solomonti04@yahoo.com)

REFERENCE

1. Lawal TA. Overview of anorectal malformations in Africa. Frontiers in surgery. 2019;6:7
2. Cassina M, Leon FF, Ruol M, Chiarenza SF, Scirè G, Midrio P, et al. Prevalence and survival of patients with anorectal malformations: A population-based study. Journal of Pediatric Surgery. 2019;54(10):1998-2003..
3. Kluth D, editor Embryology of anorectal malformations. Seminars in pediatric surgery; 2010: Elsevier
4. Hasan Z, Thakur VK, Yadav R, Chaubey D. Pattern of anorectal malformation in a tertiary care hospital of Bihar. International Surgery Journal. 2020;7(8):2647-51.
5. Mohammed M, Amezene T, Tamirat M. Intestinal obstruction in early neonatal period: A 3- year review of admitted cases from a tertiary hospital in ethiopia. Ethiopian Journal of Health Sciences. 2017;27(4):393-400.
6. Herman RS, Teitelbaum DH. Anorectal malformations. Clinics in perinatology. 2012;39(2):403-22.
7. Carter TC, Kay DM, Browne ML, Liu A, Romitti PA, Kuehn D, et al. Anorectal atresia and variants at predicted regulatory sites in candidate genes. Annals of human genetics. 2013;77(1):31- 46..
8. van de Putte R, Wijers CH, de Blaauw I, Marcelis CL, Sloots CE, Brooks AS, et al. Previous miscarriages and GLI2 are associated with anorectal malformations in offspring. Human Reproduction. 2017;32(2):299-306..
9. Zwink N, Jenetzky E, Brenner H. Parental risk factors and anorectal malformations: systematic review and meta-analysis. Orphanet journal of rare diseases. 2011;6(1):1-17.
10. Kapapa M et al., Risk factors for anorectal and associated malformations in German children: A 10- year analysis, Pediatrics and Neonatology, https://doi.org/10.1016/j.pedneo.2020.09.008 61. Oh C, Youn JK, Han JW, Yang HB, Kim HY, Jung SE. Analysis of Associated Anomalies in Anorectal Malformation: Major and Minor Anomalies. J Korean Med Sci. 2020 Apr 13;35(14):e98. doi: 10.3346/jkms.2020.35.e98. PMID: 32281315; PMCID: PMC7152527.
11. van Rooij IA, Wijers CH, Rieu PN, Hendriks HS, Brouwers MM, Knoers NV, et al. Maternal and paternal risk factors for anorectal malformations: A Dutch case‐control study. Birth Defects Research Part A: Clinical and Molecular Teratology. 2010;88(3):152-8.
12. Wang C, Li L, Cheng W. Anorectal malformation: the etiological factors. Pediatric surgery international. 2015;31(9):795-804
13. Marcelis C, de Blaauw I, Brunner H. Chromosomal anomalies in the etiology of anorectal malformations: a review. American Journal of Medical Genetics Part A. 2011;155(11):2692-704
14. Taye M, Afework M, Fantaye W, Diro E, Worku A. Factors associated with congenital anomalies in Addis Ababa and the Amhara Region, Ethiopia: a case-control study. BMC pediatrics. 2018;18(1):1-11.
15. Yuan P, Okazaki I, Kuroki Y. Anal atresia: effect of smoking and drinking habits during pregnancy. Japanese journal of human genetics. 1995;40(4):327-32
16. Rollins MD, Bucher BT, Wheeler JC, Horns JJ, Paudel N, Hotaling JM. Healthcare Burden and Cost in Children with Anorectal Malformation During the First 5 Years of Life. The Journal of Pediatrics. 2022;240:122-8. e2
17. Svenningsson A, Gunnarsdottir A, Wester T. Maternal risk factors and perinatal characteristics of anorectal malformations. Journal of Pediatric Surgery. 2018;53(11):2183-8.
18. Zwink N, Jenetzky E. Maternal drug use and the risk of anorectal malformations: systematic review and meta-analysis. Orphanet journal of rare diseases. 2018;13(1):1-23
19. Wijers CH, van Rooij IA, Marcelis CL, Brunner HG, de Blaauw I, Roeleveld N. Genetic and nongenetic etiology of nonsyndromic anorectal malformations: a systematic review. Birth Defects Research Part C: Embryo Today: Reviews. 2014;102(4):382-400
20. Stoll C, Alembik Y, Roth MP, Dott B. Risk factors in congenital anal atresias. Ann Genet. 1997;40(4):197-204. PMID: 9526612.
21. Hvide, H.K., Johnsen, J. & Salvanes, K.G. Parental age and birth defects: a sibling study. Eur J Epidemiol 36, 849–860 (2021). <https://doi.org/10.1007/s10654-021-00734-8>
22. Pethő B, Mátrai Á, Agócs G, Veres DS, Harnos A, Váncsa S, Bánhidy F, Hegyi P, Ács N. Maternal age is highly associated with non-chromosomal congenital anomalies: Analysis of a population-based case-control database. BJOG. 2023 Sep;130(10):1217-1225. [https://doi.org/ 10.1111/1471-0528.17461.](https://doi.org/10.1007/s10654-021-00734-8)
23. Hall, J.A., Benton, L., Copas, A. et al. Pregnancy Intention and Pregnancy Outcome: Systematic Review and Meta-Analysis. Matern Child Health J 21, 670–704 (2017). [https://doi.org/10.1007/s10995-](https://doi.org/10.1007/s10995-016-2237-0) [016-2237-0](https://doi.org/10.1007/s10995-016-2237-0)
24. Myers MF, Li S, Correa-Villaseñor A, Li Z, Moore CA, Hong SX, et al. Folic acid supplementation and risk for imperforate anus in China. American journal of epidemiology. 2001;154(11):1051-6.
25. *Mohammed A.A, Abdulmohsen H. A, Hamdi H. et al.* Risk factors associated with anorectal malformations development. Saudi Med J. 2020; 41 (2):157-1
