# Supplementary material for: Anorectal Malformations (ARM) and associated maternal factors among children at Tikur Anbessa Specialized Hospital and St. Paul’s Hospital Millennium Medical College, Addis Ababa, Ethiopia: An unmatched case-control study
Source: PLoS One. 2024 Sep 20;19(9):e0309298. doi: 10.1371/journal.pone.0309298 (PMC11414888; doi:10.1371/journal.pone.0309298)
Supplement: Attachment — Submitted filename: review report.docx [file pone.0309298.s006.docx]

Date: 29/04/2024

PLOS ONE

Review report

Title: Maternal risk factors associated with anorectal malformation at selected governmental

hospitals in Addis Ababa, Ethiopia, 2023: A case–control study

I. General comments

The work you have done is very substantial for evidence based birth defects, particularly in the context of anorectal malformations. However, I believe that certain adjustments are necessary to correct editorial and grammatical issues, as well as specific suggestions in each part.

II. Specific comments

1. Title

In my suggestion, title should be like “Anorectal Malformation and Associated Maternal Factors among Children at Tikur Anbessa Specialized Hospital and St Paul's Hospital Millennium Medical College , Addis Ababa, Ethiopia: Unmatched case–control study”.

2. Abstract

The introduction part of the abstract should be rewrited. It is so long. Make it short and concise. Instead of …. “Involving the lowest portion of the intestinal and urogenital tracts” rewrite as “….involving the rectum, distal anus and related urogenital tracts”. Dear authors in lines 7-8: instead of ‘embryonic development’ please say “embryo-fetal development”.

Dear authors, the objective of the study should be written as “this study aimed to assess maternal factors associated with anorectal malformation …………….” Dear authors, please do not use abbreviations in the abstract if necessary define it first. For instance what is ARM? Lines 22-23: it says “…..and then exported into SPSS version 26 for management and analysis” please replace “management and” by “statistical”. Dear authors, no need of “Both descriptive and analytical statistics was used” please delete it (line24). Dear authors, please write your major findings. No need of the present study, according to the study, etc. please delete “The present study showed the presence of a statistically significant association between advanced maternal age (age > 35 years) and birth of newborns with ARM. According to the study” (Lines 30-31). Please delete in conclusion-line -45. Line-47 say “significantly increased” instead of “appeared to increase”. Please delete “ARM” from your keywords.

3. Introduction

Dear authors, please use citation (line55). Line 57-58 “Depending on the level of disturbance”, this phrase is not clear. Please rephrase it. Lines 62-64- it says “many literature in Africa”, but you cite only one reference, please add more evidences. Dear authors, lines 65-67 have no reference, please cite. Please independently cite for each factors that contribute for anorectal malformations (lines 69-72). Please say “thus, this study aimed to assess maternal factors associated with anorectal malformations……” (Lines 73-75).

4. Methods

Please, use “methods” instead of “methods and subjects”, Dear authors, please rewrite lines 80-84. Dear authors, please use “subheadings” like study area and period, study population, sample size determination and sampling procedure, data collection and data quality assurance and statistical analysis. Dear authors, please carefully address and scientifically rewrite the “methodology” section with subheadings.

5. Results

Dear authors, please check grammar and language errors of the “result” section. Please put the tables next to citations or below the paragraph of narrated table.

6. Discussions

Dear authors, please add “a preamble paragraph” about anorectal malformation for the clarity of the whole discussion of readers. Please discuss each of the significant maternal factors of anorectal malformation each paragraph. Please give plausible evidence of narration of agreed and disagreed of findings. Please add limitation of the study in the last paragraph of the “discussion” section.

7. Conclusions

Dear authors, no need of “Several studies were conducted on the risk factors of ARM. However, these studies are inconsistent and heterogeneous. In this study it was found that” please delete it. Simply state the major findings. Dear authors, it is better to add “a recommendation statement” at the end of your conclusion.

Decision: major revision

Thank you for your effort.
