# Supplementary material for: Anorectal Malformations (ARM) and associated maternal factors among children at Tikur Anbessa Specialized Hospital and St. Paul’s Hospital Millennium Medical College, Addis Ababa, Ethiopia: An unmatched case-control study
Source: PLoS One. 2024 Sep 20;19(9):e0309298. doi: 10.1371/journal.pone.0309298 (PMC11414888; doi:10.1371/journal.pone.0309298)
Supplement: Attachment — Submitted filename: REBUTAL LETTER FOR ACCADEMIC EDDITOR.docx [file pone.0309298.s007.docx]

**AUTHOR’S RESPONSE TO ACCADEMIC EDITOR COMMENT**

**Dear editor**

I want to begin by truly appreciating your amazing, inspiring, and motivating comments on our manuscript. We also value your insightful and beneficial comments and suggestions, which we feel will help our manuscript to be of higher quality. The following changes have been made in response to your feedback.

**INTRODUCTION**

- Include more details on the specific knowledge gaps or limitations of previous studies that this study aims to address.

**Answer** - Included in more detail as commented. The previous research studies performed at Tikur Anbessa Specialized Hospital (TASH) and Jimma University Hospital had solely focused on investigating and quantifying the overall burden the medical condition in question. However, up until this point, no studies have been conducted to explore or identify the potential underlying determinants or associated factors that may contribute to the development and occurrence of this particular condition.

- Include the research objectives more clearly, perhaps as a separate paragraph at the end of the introduction.

**Answer**- Comment accepted. Included in more detail as a separate paragraph at the end of the introduction section

**METHODS:**

- The study design, setting, and sampling approach are clearly described. It would **be good** to provide **more details** on how the sample size was calculated, such as ***the expected effect size***, power, and significance level used.

**Answer** – comment accepted and corrected accordingly

- The data collection and analysis methods **are well-explained**. It would be useful to **include information** on **how anorectal malformations were diagnosed** and classified in this study.

**Answer** - The materials and methods section of the paper discusses the process used to diagnose anorectal malformations (ARM) in the study participants.

However, as the focus of this research project was not on examining the specific patterns or classifications of the different types of ARM, it may not be necessary to provide a detailed discussion of ARM classification schemes within the methods section, as that information may not be directly relevant to the primary goals of the study. Instead, the introduction section of the paper already included a thorough overview of the most up-to-date classification system for anorectal malformations.

- The statistical analysis plan is comprehensive, but it would be helpful to explain the **rationale for using both bivariate and multivariate analyses**.

**Answer** – comment accepted and corrected accordingly. The rationale for using bivariable binary logistic regression and multivariable logistic regression was discussed in the revised manuscript in detail.

**RESULTS**

- The results are **presented clearly and the key findings are highlighted**. It would be good to **include** a table summarizing the demographic and clinical characteristics of the study participants.

**Answer** - Comment accepted. I included one additional table showing the

Pregnancy and obstetric characteristics of the study participants.

- The interpretation of the results, particularly **the adjusted odds ratios**, could be strengthened by discussing the **plausibility and potential mechanisms underlying the observed associations.**

**Answer -** The strength of association between the dependent and independent variables was discussed with reference to the AOR. The discussion section provides a good synthesis of the key findings and compares them to existing literature.

- It would be valuable to **discuss** the strengths and limitations of the study design and how they may have influenced the results.

**Answer** - Comment accepted. Limitations related to the study design like recall

Bias and lack of power were included in the revised manuscript.

- The concluding remarks could be expanded to highlight the public health implications of the findings and propose future research directions.

**Answer -** Included as commented

**Overall:**

The manuscript is generally well-written and organized. Consider improving the flow between sections and ensuring consistent terminology (e.g., "ARM" vs. "anorectal malformation").

Check the formatting and ensure all references are complete and correctly cited. In summary, this is a well-designed and conducted study that contributes to the understanding of maternal risk factors for anorectal malformations in the Ethiopian context. With some revisions to strengthen the introduction, methods, results interpretation, and discussion, this manuscript would be a valuable addition to the literature.

**.** Be sure to:

- Indicate which changes you require for acceptance versus which changes you recommend
- Address any conflicts between the reviews so that it's clear which advice the authors should follow
- Provide specific feedback from your evaluation of the manuscript.

**Feedback**

We thoroughly reviewed and updated the entire manuscript. We carefully considered and incorporated each specific suggestion and comment provided by the manuscript reviewers. We found the feedback from the reviewers to be extremely valuable and beneficial. The reviewer comments significantly improved the overall quality and structure of the final manuscript.
